# Editorial: Frontiers in Dental Medicine: highlights in regenerative dentistry 2021/22

**DOI:** 10.3389/fdmed.2023.1212894

**Published:** 2023-05-26

**Authors:** Waruna Lakmal Dissanayaka, Paul Sharpe

**Affiliations:** ^1^ Applied Oral Sciences and Community Dental Care, Faculty of Dentistry, The University of Hong Kong, Hong Kong, Hong Kong SAR, China; ^2^ Centre for Craniofacial and Regenerative Biology, Faculty of Oral and Craniofacial Sciences, Guy’s Hospital, King’s College London, London, United Kingdom

**Keywords:** regenerative dentistry, hydrogel, wnt signaling, tooth development, enamel regeneration

**Editorial on the Research Topic** Frontiers in Dental Medicine: highlights in regenerative dentistry 2021/22

According to the global oral health status report issued by WHO for 2022, oral diseases are the most widespread noncommunicable diseases affecting almost half of the world's population (45% or 3.5 billion people worldwide) over the life course from early life to old age. The traces of the prevalence of caries and periodontal disease run into ancient times, but the complete rehabilitation of caries or periodontal disease-affected tissues is yet to be achieved. Current treatment options offer to control the disease process to obtain symptomatic relief or/and restoration of tissue by artificial materials. As adult human teeth are not replaced naturally, it is necessary to find ways to achieve a fully functional recovery of teeth affected by oral diseases and conditions. Regenerative dentistry aims to regenerate injured or diseased tissue or the whole functional dental organ using biologically based approaches. The current Research Topic highlights some of the important advancements in regenerative dentistry.

Tooth development is a very important area of research that provides valuable insight into regenerative dentistry. Studying tooth development is necessary for unraveling the molecular mechanisms in development and using this knowledge in tooth regeneration. For example, Wnt signaling is one of the important pathways in tooth development. The importance of Wnt signaling in proper tooth development is emphasized in two review articles in the Research Topic that together, comprehensively, cover its role in tooth development, root formation, periodontal ligament, and bone, concluding with potential clinical applications of the modulation of this pathway. In the review by Tokavanich et al. they emphasized that the proper expression of Wnt signaling during dental development is crucial for regulating cellular proliferation and differentiation as well as the epithelial-mesenchymal interaction essential for tooth root formation and tooth eruption. Accordingly, Wnt signaling plays a major role in the response of odontoblasts to injury and repair processes. It is also directly linked to the healing and repair process that is fundamental to recovering oral tissues affected by the inflammatory process and recovery for regeneration. The review by Birjandi and Sharpe from Kings College London explores how the Wnt signaling pathway could be utilized to enhance regenerative approaches by promoting healing and repair.

Biomaterials play a major role in carrying the signaling mediators or cells to the target host tissue. Various biomaterials encapsulated with biomolecules or cells have been utilized in regenerative approaches and demonstrated positive results *in vitro* and *in vivo*. Some of these approaches are currently in phase I/II clinical trials on evaluating their safety and efficacy. In our Research Topic, groups from the University of Southern California and Johns Hopkins University, Baltimore, presented a study where amelogenin-derived peptides P26 and P32 incorporated chitosan hydrogel that was fabricated for the remineralization of the enamel, which is a fundamental requirement for the regeneration of a complete tooth structure. Mukherjee et al. reported that P26 peptide alone or in combination with chitosan significantly enhanced the remineralization of the enamel. The ability to regrow tooth enamel could be a major step in the functional recovery of the tooth structure.

Various enhancements in the field of regenerative dentistry have raised enthusiasm among clinical dental practitioners to utilize these advances in their practice. However, the study conducted by Neves and Jamal from Kings College London among practicing dentists in the UK demonstrated that there is a bigger knowledge gap between clinicians and researchers on the potential applications of regenerative dentistry. As a result, some dentists are still skeptical about applying these therapies in the clinical field which emphasizes the need to transfer knowledge from benchtop to the clinic. Most dentists have the desire to educate themselves more on the advances of regenerative dentistry and how they could utilize them in their practice. Therefore, it is the need of the hour of the regulatory and educational bodies to corporate and make a clear consensus to enhance the knowledge of regenerative dentistry among dentists as well as dental students.

